# Break-Induced Replication and Genome Stability

**DOI:** 10.3390/biom2040483

**Published:** 2012-10-16

**Authors:** Cynthia J. Sakofsky, Sandeep Ayyar, Anna Malkova

**Affiliations:** Department of Biology, School of Science, IUPUI, Indianapolis, Indiana 46202, USA; Email: csakofsk@iupui.edu (C.J.S.); sandeepayyar19@gmail.com (S.A.)

**Keywords:** double-strand break (DSB), DNA repair, break-induced replication (BIR), recombination

## Abstract

Genetic instabilities, including mutations and chromosomal rearrangements, lead to cancer and other diseases in humans and play an important role in evolution. A frequent cause of genetic instabilities is double-strand DNA breaks (DSBs), which may arise from a wide range of exogeneous and endogeneous cellular factors. Although the repair of DSBs is required, some repair pathways are dangerous because they may destabilize the genome. One such pathway, break-induced replication (BIR), is the mechanism for repairing DSBs that possesses only one repairable end. This situation commonly arises as a result of eroded telomeres or collapsed replication forks. Although BIR plays a positive role in repairing DSBs, it can alternatively be a dangerous source of several types of genetic instabilities, including loss of heterozygosity, telomere maintenance in the absence of telomerase, and non-reciprocal translocations. Also, mutation rates in BIR are about 1000 times higher as compared to normal DNA replication. In addition, micro-homology-mediated BIR (MMBIR), which is a mechanism related to BIR, can generate copy-number variations (CNVs) as well as various complex chromosomal rearrangements. Overall, activation of BIR may contribute to genomic destabilization resulting in substantial biological consequences including those affecting human health.

## 1. Introduction

Genomic instability leading to mutations and chromosomal rearrangements can enable pre-cancerous cells to acquire many hallmarks of cancer, such as limitless replicative potential, evasion of cell death and constant proliferation. Although no clear explanation for the origin of genetic instability exists, the activation of oncogenes in pre-cancerous cells have been found to lead to over-initiation of replication which results in the collapse of replication forks [1,2,3,4], the accumulation of DNA breaks, and a burst of genetic instability [1,3,4,5,6]; reviewed in [7,8]. In particular, genetic instability has been linked to DSBs formed from collapsed replication forks. BIR is a DSB repair mechanism capable of recovering collapsed replication forks that is known to produce genomic rearrangements and genetic mutations at high frequencies. BIR, which was originally discovered and investigated in bacteria and viruses, has recently been thoroughly studied using yeast *Saccharomyces cerevisiae*, a model eukaryotic organism. Studies in yeast have provided details of molecular mechanisms, regulation, and proteins participating in BIR, as well as analyses of genetic instabilities resulting from BIR. BIR has yet to be studied in mammals, although some evidence has suggested that it operates in humans, and that its activation is a probable cause of many human diseases. In this review, we use the data obtained from yeast to discuss mechanisms of BIR and to explain the reasons for genetic instabilities resulting from this DNA repair pathway. In addition, the history of BIR investigation in prokaryotes, called recombination-dependent replication (RDR) in bacteria and viruses, is also briefly described. The reader however is referred to other recent reviews [9,10,11,12,13,14] for more detailed information on RDR in bacteria and viruses. Furthermore, we examine recent evidence which connects microhomology-mediated BIR (MMBIR), a BIR-related mechanism, to human diseases.

## 2. Double-Strand Break Repair Mechanisms

**Figure 1:**
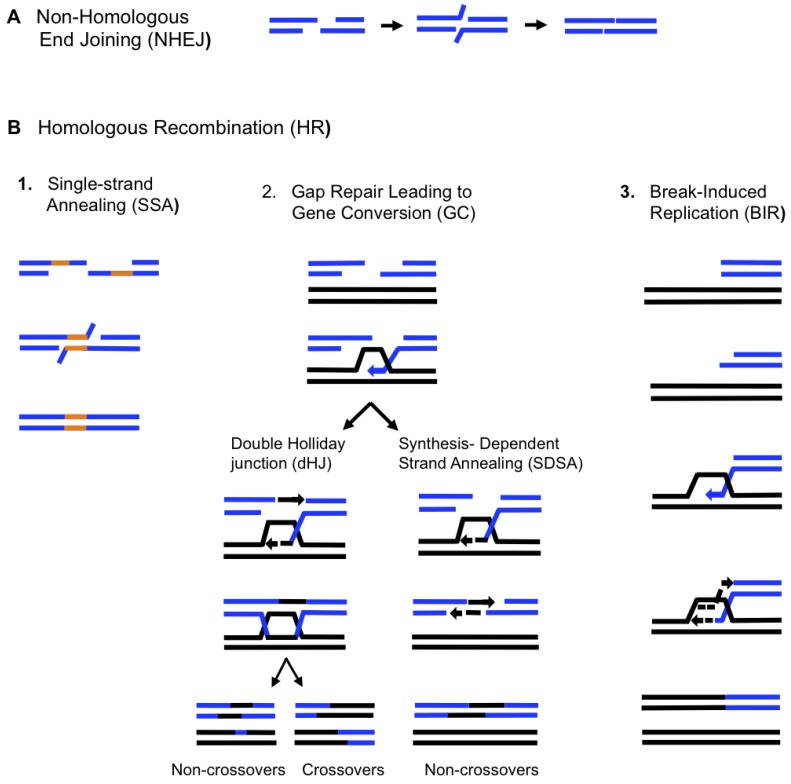
Mechanisms of double-strand DNA breaks (DSB) repair. (A). Non-homologous end joining (NHEJ) involves re-ligation of broken DNA ends resulting in small deletions or insertions (B). Repair by homologous recombination (HR) starts with resection of DSB ends in a 5’ to 3’ direction and requires the involvement of long homologous regions. Repair by HR can proceed by 3 different pathways, Single Strand Annealing (SSA), Gap Repair leading to gene conversion (GC) and Break-Induced Replication (BIR). (1) SSA occurs through annealing of DNA direct repeats (orange lines) after they become single-stranded. Annealing leads to the deletion of all sequences between the repeats and to the loss of one repeat [37] (2) Gap repair leading to GC. GC may proceed via the formation of a double Holliday Junction (dHJ), leading to the formation of crossover products about half of the time [18]. GC may also result from SDSA, which does not involve the formation of a dHJ and rarely leads to crossover products [19]. In all cases, GC is associated with a short patch of DNA synthesis. (3) BIR is initiated by a one-ended DSB and proceeds via copying of large DNA regions [33,38,39]. Dashed lines indicate newly synthesized DNA.

Double-strand DNA breaks (DSBs) are potentially lethal events that can occur from exposure to DNA-damaging agents such as radiation, various chemicals and anti-cancer drugs, but they can also be formed spontaneously as a result of problems with DNA metabolism (*i.e.*, problems during replication and segregation) (reviewed in [15,16]). Two major pathways, homologous recombination (HR) and non-homologous end-joining (NHEJ), have evolved to repair DSBs. NHEJ proceeds by the interaction of broken DNA ends that are not homologous to each other or contain micro-homologies (Figure 1A). NHEJ frequently leads to deletions and insertions and therefore can be mutagenic [17]. HR involves interactions between large homologous DNA regions and can proceed through several pathways including single-strand annealing (SSA), gap repair leading to gene conversion (GC), and break-induced replication (BIR) (Figure 1B). SSA repairs DNA breaks that are flanked by long direct DNA repeats (Figure 1B.1). SSA is initiated by 5’-to-3’ DNA resection, which renders direct repeats single stranded allowing them to anneal to each other. This process leads to a loss of DNA between the repeats and a reduction of the repeats to a single copy. GC proceeds by the invasion of one broken 3’ DNA end into a homologous template, followed by copying of the DNA sequences necessary to repair the break; the second 3’ end of the break either anneals with the single-stranded DNA in the D-loop (forming a double Holliday junction, dHJ) [18], or anneals with the extended and displaced 3’ end that initiated strand invasion (in synthesis dependent strand annealing, SDSA) [19] (Figure 1B.2). 

GC leads to the repair of the DSB by a limited patch of new DNA. GC has traditionally been considered the ‘safest’ pathway of DSB repair because it is rarely associated with genetic rearrangements, though mutations have been associated with this type of repair [20,21]. Further details on NHEJ, SSA and GC repair pathways are reviewed in [15,16,22]. When homology between the donor and recipient chromosomes is restricted to a single side of a DSB, GC is abolished and the repair of such one-ended breaks proceeds by BIR (Figure 1B.3). According to existing models, BIR proceeds through the invasion of one broken DNA end into an intact donor chromosome followed by the initiation of DNA synthesis that can proceed to the end of the donor chromosome (reviewed in [23,24]). BIR plays an important role in the repair of one-ended breaks that can be formed as a result of collapsed replication forks and eroded telomeres. Repair by BIR can be dangerous for a cell however, because it can result in the copying of hundreds of kilobases of DNA from a donor molecule while a large piece of broken unrepaired DNA can be lost. Also, BIR can lead to various types of genomic instabilities, including mutations and chromosomal rearrangements [25,26,27,28,29,30,31,32,33,34,35,36].

## 3. Recombination-Dependent Replication (RDR) in Bacteriophages and Prokaryotes

BIR was first identified in the late replication phase of bacteriophage T4 where it was described as RDR [40]; reviewed in [41]. In addition to its role in late replication, RDR in bacteriophage T4 was shown to play important roles in DSB repair and in the repair of broken replication forks [42,43,44] (see also [9,11,41,45] and references therein). RDR was also found to be involved in several different processes in *Escherichia coli*, including stable DNA replication [46,47], (see also [48] and references within), repair of double-strand DNA breaks [46,47,49], repair of collapsed replication forks (see in [13,14] and references therein), and a process leading to the formation of adaptive mutations [50,51]. It was demonstrated that similar groups of proteins are required for BIR in bacteria and phages including recombination proteins, replication proteins, and proteins mediating recombination and replication. Recombination proteins initiate the BIR process by promoting strand invasion and D-loop formation [47,52,53,54], also see in [9,11]. The role of mediator proteins is to assemble a processive replication fork on the D-loop that is formed during the first step of BIR. This function is carried out by the PriA complex or by PriC in *E. coli* (see in [48] and references within) and by the gp59 protein in bacteriophage T4 (reviewed in [9,11,55]). The critical step in this process is loading the replicative helicase that is capable of recruiting primase, thus promoting the assembly of the replication fork. During normal DNA replication in *E. coli*, the replicative helicase DnaB is loaded by DnaA, which interacts with the bacterial origin of replication OriC. During BIR, the origin recognition step is replaced by the recognition of the D-loop by a PriA complex (which includes PriA, PriB and DnaT) or by PriC, which promotes loading of DnaB (reviewed in [13,48,56]). DnaG primase subsequently interacts with DnaB, completing the assembly of a primosome. A similar sequence of events takes place in T4 phage, where gp59 plays the role of PriA (reviewed in [9,55]). (Note: We refer the reader to reviews for details on the PriA complex and T4 phage gp59 protein, as much literature regarding these key recombination factors has been published). The last replication stage of BIR is carried out by processive DNA polymerases working in conjunction with clamp and clamp-loader proteins (polymerase III complex in *E. coli*) [57,58] and gp43/gp/44/gp45/gp62 complex in T4 (reviewed in [9]. Among all listed proteins, “mediators” are the only ones that are truly unique for RDR because they carry out a unique function by providing a link between recombination and replication. It was demonstrated that PriA and PriC initiate RDR by binding single-stranded (ss) DNA that was formed in the vicinity of collapsed replication forks (see in [13,59] and references within). Interestingly, Pri complexes were shown to initiate RDR at R-loops, when they were stabilized in the absence of RNaseH destroying RNA/DNA hybrids [60]. The details of the molecular mechanism of RDR and proteins participating in RDR can be found in several reviews that are specifically focused on RDR [9,11,13,48,59].

## 4. BIR in Eukaryotes

BIR is well studied in the yeast *Saccharomyces cerevisiae.* Several specific experimental systems have been developed in this organism to study BIR. One approach uses yeast transformation, where a linearized fragment containing a centromere is used to initiate BIR [38]. In particular, a chromosome fragmentation vector (CFV) is linearized by a restriction endonuclease prior to transformation into yeast. Once in the cell, one end of the vector acquires a *de novo* telomere, while the other end invades a homologous region of chromosomal DNA, thus initiating BIR. The use of this system by Morrow *et al.* [38], and later by Davis and Symington [33] allowed the demonstration that BIR could synthesize hundreds of kilobases of DNA. Recently, a modified version of this system allowed yeast cells to be transformed with CFVs containing an *I-SceI* endonuclease recognition site [61]. This vector remains intact within the cell as an episome until the addition of galactose, which will linearize the vector and initiate BIR. 

Another approach makes use of HO endonuclease, which initiates a DSB in such a way that only one broken DNA end can find homology in the yeast genome, thus resulting in repair by BIR. Using HO-induced DSBs, Bosco and Haber [26] documented BIR in a situation where the invading strand and the template DNA shared only 70 base pairs of homology. The generation of DSBs by HO in diploid and partially diploid cells has been used to investigate allelic BIR. This was accomplished by truncating one of two copies of chromosome III in such a way that only one DSB end could invade a homologous region, thereby being repaired by BIR [27,39]. Also, ectopic BIR has been studied in a yeast haploid system where an HO cut site was positioned on chromosome V. Upon DSB induction, one broken DSB end invaded a homologous region on chromosome XI that initiated BIR and led to a translocation [34]. A similar experimental system utilized by Ruiz *et al*. [32] also allowed the investigation of ectopic BIR. 

The study of BIR in higher eukaryotes has been limited thus far, though recently one experimental system utilizing *Xenopus laevis* egg extracts has been effective in examining cellular responses to nicked DNA during replication [62]. The occurrence of a nicked DNA template during replication fork progression generates a one-ended DSB, which is an ideal substrate for BIR. 

BIR has yet to be studied systematically in mammals, primarily due to an absence of a reliable experimental system. Nevertheless, different genetic instabilities that lead to cancer in mammals, such as loss of heterozygosity (LOH) (reviewed in [63,64,65]) and the formation of chromosomal translocations (reviewed in [66,67,68,69]) may result from BIR. In addition, BIR is likely to be responsible for alternative telomere lengthening (ALT), a recombination-based mechanism that is often used by telomerase-compromised cancer cells to maintain long telomeres. ALT proceeds by the invasion of uncapped telomeres into telomere or sub-telomere regions of other chromosomes followed by copying which allows the extension of telomeres in the absence of telomerase (reviewed in [24,70,71,72,73,74,75]). Studies in yeast have demonstrated that ALT proceeds using BIR [34]. 

## 5. Molecular Mechanism of BIR

### 5.1. Initiation of BIR

**Figure 2:**
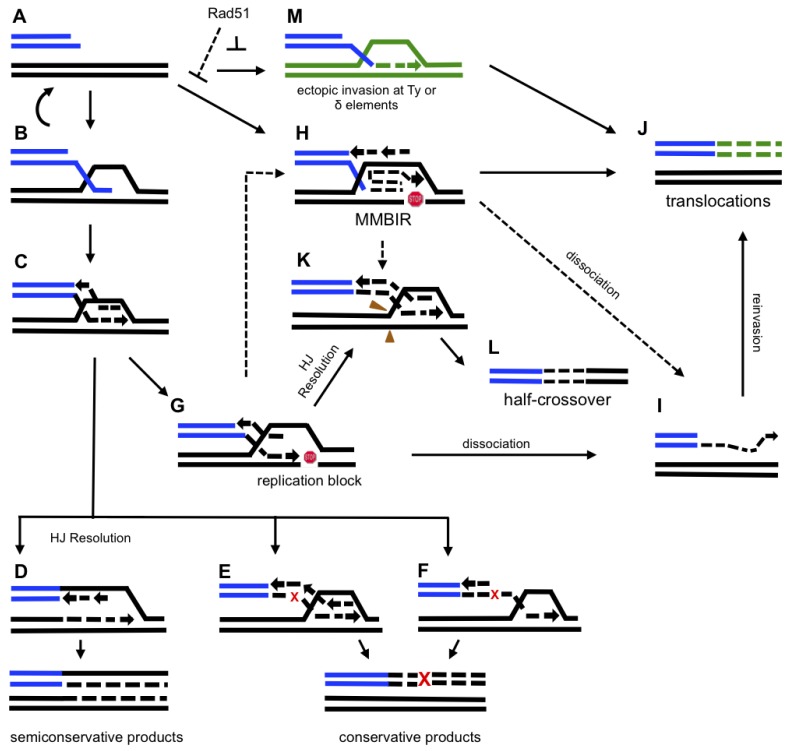
Model for BIR-induced genetic instability. A schematic indicating proposed pathways of BIR-induced genetic instability. Abbreviations: HJ, Holliday junction; MMBIR, micro-homology mediated BIR. A small red “x” indicates an error during BIR which leads to a fixed mutation, as indicated by a large red “X”. A red “stop” symbol indicates stalling of BIR replication. Green color indicates non-homologous chromosomes. **A.** 5’ to 3’ resection of a one-ended DSB. **B.** 3’ overhang invasion into a homologous chromosome. **C.** 3’ strand invasion leading to the formation of a unidirectional replication fork. **D.** Replication via semi-conservative DNA synthesis. **E and F.** Conservative replication associated with branch migration occurring by **(E)** coordinated synthesis of leading and lagging strands or **(F)** initial leading strand synthesis, later serving as the template for lagging strand synthesis. **G.** A pause during BIR replication leading to one of the following outcomes **(H-L)**: (i) **H.** a switch to MMBIR; (ii) **I.** dissociation of a newly synthesized strand; (iii) **J.** a translocation resulting from strand invasion into a non-homologous chromosome; (iv) **K.** the processing of BIR intermediates; (v) **L.** a half-crossover resulting from **(K).M.** 3’ end invasion at an ectopic position which leads to a translocation **(J)**. Letters connected by dotted lines denote the following hypothetical events: (i) a switch from BIR to MMBIR **(H),** initiated by pausing of BIR **(G)**; (ii) MMBIR **(H)** leads to half-crossovers **(K)** and **(L)**; and (iii) MMBIR **(H)** leads to strand dissociations **(I)** which then results in translocations **(J)**.

Studies in yeast revealed the following details about the mechanisms associated with BIR. BIR is initiated by a DSB that undergoes an extensive 5’-3’ resection [76], resulting in long 3’ single-stranded DNA ends which invade homologous DNA sequence forming a D-loop (Figure 2A, 2B). Strand invasion depends on Rad52 (Table 1) [33,77], and can proceed through either Rad51-dependent [33,39] or Rad51-independent pathways [28,77]. The most efficient pathway, Rad51-dependent, also requires Rad55, Rad57 and Rad54 [78]. For high efficiency, Rad51-dependent BIR requires significant (>1kb) regions of homology between interacting DNA molecules [34]. The Rad51-independent pathway is not very efficient and depends on Rad59, the Rad54 homolog Tid1, and the Mre11-Rad50-Xrs2 complex [78]. Rad51-independent BIR has been described in yeast diploids when HO-induced DSBs were introduced into the middle of one copy of chromosome III [77]. A striking feature of BIR occurring in the absence of Rad51 is that generally it leads to translocations (Figure 2A, 2M, 2J) resulting from strand invasion of a broken chromosome at ectopic positions at locations of Ty and delta elements [28]. In contrast, strand invasions into allelic positions are very rare. Downing *et al*. [79] proposed that the absence of Rad51 stimulates the initiation of BIR at regions containing relatively small homology to the broken DNA end and that BIR might be mediated by annealing of the broken end to regions that became single-stranded due to activation of replication, transcription or formation of secondary DNA structures in the donor chromosome. 

**Table 1:** Requirements for recombination and replication proteins in Gap Repair (Gene Conversion) and BIR in yeast.

Proteins	Requirement of the protein in the repair pathway	
	Gap repair (Gene Conversion)	BIR*
Rad52	Required [15,86,87]	Required [33,77]
Rad51	Required [15,86,87]	Required [33,39]
Rad55, Rad57	Required [15,86,87]	Required [78]
Rad54	Required [15,86,87]	Required [78,79]
Pol32	Not required [34] / required **[31]	Required [27,29,34]
Mcm 2-7	Not required [88]	Required [89]
PCNA	Required [88,90]	Required [89]
Polα/Primase	Not required [88]	Required [34]
Polδ	One is required (Polδ and Polε can substitute each other) [90]	Required [27,29,34]
Polε		Required***[34]
Cdc45	Not required [88]	Required [89]
GINS, Cdt1, Cdc7	?	Required [89]
Cdc6	?	Not required [89]
ORC	Not required [90]	Not required [89]

* Protein requirements for *RAD51*-dependent BIR are included

** Pol32 is required for repair of large gaps [31]

*** Approximately 25% of BIR can be completed in the absence of Pol [34].

Question marks represent cases where the requirement for corresponding proteins in gap repair is unknown.

The requirements of Rad51-dependent and Rad51-independent ALT in yeast correlate with the requirements for BIR [80,81]. For example, Rad51-dependent ALT requires Rad52, analogous to the Rad51*-*dependent BIR pathway, and leads to the proliferation of long, sub-telomeric Y' and other sub-telomeric sequences at nearly all chromosome ends [81,82]. Rad51-independent ALT also requires Rad52, however, similar to Rad51-independent BIR it also requires Rad59 and the MRX complex [81]. Rad51-independent ALT results in the elongation of the (TG)_n _ telomere sequences [83,84,85]. The different outcomes in the Rad51-dependent and Rad51-independent ALT probably reflect different requirements for homology between the DSB end and its template.

### 5.2. DNA Synthesis Associated with BIR

DNA synthesis during BIR has primarily been studied for Rad51-dependent BIR, and therefore we will focus on this pathway. It has been demonstrated that while the strand invasion step for BIR and GC occurred rapidly [31], these two pathways differ at the beginning of DNA synthesis. In particular, the initiation of DNA synthesis during BIR (Figure 2C) takes 3-5 hours longer as compared to GC [31,39]. This delay might result from a prolonged assembly of a BIR-specific replication fork. Alternatively, the delay in initiation of BIR DNA synthesis might result from a recombination checkpoint [31] which would specifically suppress the onset of BIR in order to create a preference for repair by GC. Additionally, frequent strand dissociation-reinvasion cycles at the beginning of BIR might be responsible for the delay and for frequent template switching observed at the beginning of BIR synthesis [30]. Importantly, after approximately 10kb of copying, BIR is stabilized and progresses at a normal pace [30,39]. 

There are several possibilities as to how DNA synthesis commences. The first possibility is that a Holliday junction (HJ) that results from strand invasion is resolved and the D-loop is transformed into a complete unidirectional *bona fide* replication fork that migrates down the template chromosome (Figure 2D). This would result in two semi-conservatively replicated molecules. Several properties of BIR observed in yeast are consistent with this idea. Thus, the observation made by Lydeard *et al*. [89], that initiation of BIR involves the majority of proteins that participate in initiation of normal S-phase DNA replication points towards a similarity between a replication fork formed during BIR and one established during S-phase replication. Also, it was observed that the rate and processivity of established BIR is similar to those during normal DNA replication [39]. The *bona fide* replication fork, however, does not explain a number of recent observations, such as an increased propensity of BIR-associated replication to generate chromosomal rearrangements, mutations and template switches [25,29,30]. Another possibility is that the HJ is not resolved at the beginning of BIR, as was also suggested by [23,30]. In this case, the HJ would be acted upon by branch-migration enzymes that displace the newly synthesized leading and lagging strands, thereby generating molecules that mimic the products of conservative replication (Figure 2E). Finally, another scenario is possible where BIR is carried out by two rounds of repair-type DNA synthesis: the first round creates a single-stranded DNA molecule that is used as a template in the second round of repair synthesis, resulting in the formation of a conservatively replicated molecule (Figure 2F). These models proposing a conservative mode of BIR DNA synthesis are more likely to account for various genetic abnormalities resulting from BIR (as previously suggested by [23,30]). 

Characterization of replication proteins participating in BIR demonstrated that initiation of BIR involves the majority of proteins that start S-phase DNA replication, including Cdc7, Cdt1, Mcm2-7, Cdc45, and GINS [89] (Table 1). These requirements make BIR different from GC, which does not require Mcm2-7 or Cdc45 [88]. BIR does not require Cdc6 and ORC, which is most likely related to the origin-independent nature of BIR [89]. Additionally, several observations indicate that the composition of proteins participating in the BIR replisome differs from that in normal DNA replication. For example, BIR requires Pol32p [27,29,34] a subunit of DNA polymerase δ that is dispensable for S-phase replication. Since Pol32 is known to mediate interactions between Polδ and other replication and repair proteins, it is possible that some other specific proteins are involved in the BIR replication fork. Furthermore, the roles of the main replicative polymerases in BIR differ significantly from their respective roles during S-phase replication. For example, while Polα and Polδ are necessary at all steps of BIR in yeast, Polε is non-essential for the initiation of BIR and is required only at later stages. In fact, 25% of BIR events can be completed in the absence of Polε [34]. These observations therefore imply that BIR replication differs from S-phase replication. 

Studies of the restart of collapsed replication forks by BIR in *Xenopus laevis* demonstrated that the key step in re-initiation of DNA synthesis occurred by a Rad51-dependent re-loading of Cdc45, GINS, and Polε [62]. Interestingly, the authors observed that Mcm2-7 does not dissociate from the replication fork following its collapse, which therefore eliminates the need for its re-loading at the beginning of BIR, which is different from the situation observed at the beginning of BIR initiated in yeast by HO endonuclease. Also, while Polη appeared to play a key role during initiation of BIR in *Xenopus,* its role in yeast BIR has not been detected thus far.

## 6. Mutagenesis Associated with BIR.

**Figure 3:**
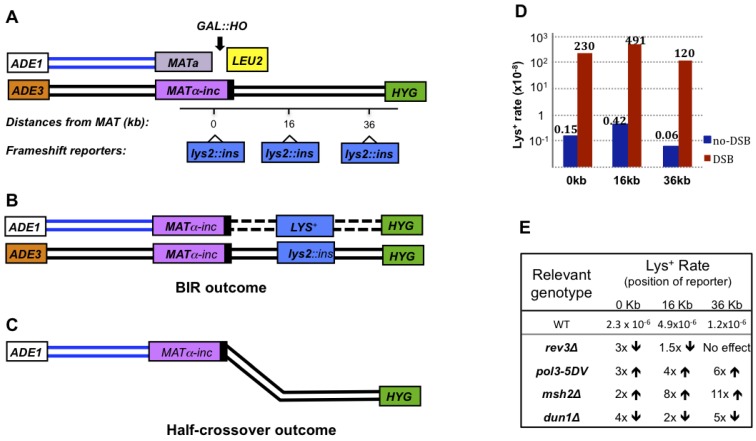
BIR is associated with increased frameshift mutagenesis. **A**. Experimental system to study BIR, half-crossovers and the level of mutagenesis associated with BIR. BIR is induced in a yeast strain disomic for chromosome III by an *HO-*induced DSB at *MAT*a of truncated chromosome III. The donor *MATα*-inc chromosome is full-length and resistant to cutting by *HO*. Frameshift reporters *lys2::ins* are integrated at three positions in the *MATα*-inc chromosome at different distances from *MATα*-inc (0, 16, or 36 kb). **B**. BIR-induced Lys^+ ^outcome. **C**. A half-crossover DSB repair outcome resulting from a fusion between the left and right portions of the broken and donor chromosomes, respectively. **D**. BIR-induced Lys^+^ mutation rates in wild-type (WT) strains [25]. BIR mutation rates exceed the level of spontaneous events (denoted as no-DSB) by approximately 1000-fold at all positions. **E**. Summary table showing the relative effects of various genetic backgrounds (*rev3Δ, pol3-5DV, msh2Δ,* and *dun1Δ)* on BIR mutation rates based on results from [25]. The “up” and “down” arrows indicate an increase and decrease in Lys^+^ mutation rates, respectively, as compared to Lys^+^ mutation rates in corresponding wild-type (WT) strains. Adapted from Deem *et al*. [25].

It is known that BIR is capable of copying large, replicon-sized chromosomal regions, which has made it important to characterize the fidelity of BIR-associated DNA synthesis. The idea that BIR could be mutagenic first came from the observation of frequent template switching occurring at the beginning of BIR [30]. In addition, Schmidt *et al*. (2010) [91] studied the formation of spontaneous translocations between homeologous genes *CAN1, LYP1* and *ALP1* and observed base substitutions and slippage events at breakpoints of some translocations. The authors explained this finding by error-prone BIR. However, direct evidence of increased mutagenesis associated with BIR came from the study of Deem *et al*. [25], where yeast strains disomic for chromosome III (Figure 3A) were used. In this study, the HO-induced DSBs were introduced into a truncated copy of chromosome III and were repaired by BIR using an uncut full size copy of chromosome III as a template (Figure 3B). The insertion of frameshift reporters at different positions in a template chromosome along the path of BIR allowed the authors to determine the frequency of frameshifts associated with BIR. The authors demonstrated that frameshifts during BIR were approximately 1000 times more frequent than during S-phase DNA replication (Figure 3D). Importantly, the increased level of frameshift mutagenesis was observed over the entire path of BIR. In addition, the authors observed that *pol3-5DV* mutation, which eliminates proofreading activity of Polδ, increased BIR-associated frameshifts. This suggested that the fidelity of Polδ is decreased during the course of BIR, which therefore explains the increase of mutagenesis (Figure 3E). The role of mismatch repair (MMR) was also investigated, with results showing a significant increase in BIR mutation rates in MMR-deficient mutants (*msh2Δ* and *mlh1Δ*). This suggested that MMR is active during BIR (Figure 3E), however MMR was less efficient during BIR than during S-phase replication. In addition, the data suggested that BIR was associated with a *DUN1*-dependent increase of dNTP pools, which contributed to an increase in mutations (Figure 3E). Together, the data provided an initial description of mutagenesis during BIR, however the underlying mechanisms involved still remain unclear. It seems that BIR mutagenesis may actually occur through multiple pathways (Figure 3E). For example, the effect of Polζ was position-dependent as it played a more prominent role in mutagenesis at the initiation of BIR than in later steps. 

Overall, it was concluded that BIR, which mimics normal DNA replication in its propensity to replicate large chromosomal regions, is substantially more mutagenic as compared to S-phase DNA replication. Importantly, recent data have suggested that the frequency of base substitutions is also highly increased during BIR (S. Ayyar and A. Malkova, unpublished observation). Thus, in respect to the fidelity of DNA synthesis, BIR appears more similar to several other pathways of DSB repair including GC and SSA that also were shown to be mutagenic [20,21,92]. However, what sets BIR apart from other DSB repair pathways is that BIR proceeds via a replication fork-like intermediate.

## 7. Chromosomal Rearrangements Associated with BIR

Besides being mutagenic, BIR frequently leads to gross chromosomal rearrangements (GCRs). Two main classes of GCRs have been described in association with BIR: translocations and half-crossovers. BIR initiated by the invasion of a broken DNA end at a non-allelic position leads to non-reciprocal translocations (Figure 2M, 2J) [26,30,31,32,33,34]. Ectopic invasions occur at positions of DNA repeats, often at locations of transposons [28,35,36], which are highly dispersed around the genome. Non-reciprocal translocations initiated by site-specific DSBs have been described in a number of BIR studies. In some of these cases [26,32,34], DSBs were initiated close to a repeated sequence, which initiated an invasion into a homologous sequence that was located at a different position in the genome. For example, Bosco and Haber [26] demonstrated that an HO-induced break introduced at *HML* initiated strand invasion at a 70-bp homologous sequence at *HMR* resulting in translocation. Lydeard *et al*. [34] showed that an HO-induced break initiated next to a *CAN1* gene on chromosome V led to a strand invasion of the *CAN1*-containing DNA end into a homologous portion of another *CAN1* gene inserted at chromosome XI, which resulted in a translocation. Several other studies in yeast described the formation of translocations mediated by BIR involving transposons: Ty and delta elements. For example, VanHulle *et al*. [28] observed that HO-induced DSBs introduced into chromosome III, 30 kb away from a pair of inverted Ty1 elements, initiated chromatid fusions that were mediated by recombination between non-allelic Ty elements. This event resulted in the formation of dicentric molecules. The mitotic breakage of these molecules led to breakage-fusion bridge cycles (BFB), and eventually to the formation of GCRs resulting from BIR events between Ty or delta elements located on the broken chromosome and at an ectopic position. Similarly, translocations introduced by *I SceI*–DSBs and mediated by Ty or delta elements located far away from the break have been described by Hoang *et al*. [36]. Importantly, these studies concluded that ectopic BIR leading to GCRs were capable of competing with allelic BIR. Furthermore, it was demonstrated that a number of mutations including *sgs1Δ* and *rad51Δ* affect competition between ectopic and allelic BIR, making ectopic GCR more favorable and GCRs more frequent [28,35,79]. Additionally, the formation of complex translocations was documented by several studies where BIR initiated by the invasion into one chromosome was first interrupted, and then continued by reinvasion and copying from another donor template [28,30].

Other studies have described spontaneous non-reciprocal translocations that occurred by BIR. Petes’ group demonstrated that reducing the levels of Polα or Polδ led to chromosomal breakage close to a pair of inverted Ty1 elements which resulted in GCRs through interactions between Ty or delta elements on the broken chromosome and those located ectopically [93,94]. Similarly, Lobachev’s group observed frequent chromosome breaks at positions of inverted DNA repeats leading to translocations that were explained by BIR mediated via Ty or delta elements [95]. An extensive study that was undertaken by Kolodner’s group, led to the discovery of many mutations stimulating the formation of GCRs through several different pathways including BIR [96,97]. For example, Schmidt and Kolodner demonstrated that *sgs1Δ* stimulated the formation of complex translocations resulting from BIR involving several diverged genes [96]. The structural analysis of these translocations indicated the involvement of breakage-fusion-bridge cycles and template switching [91].

Another type of GCRs associated with BIR is a half-crossover, (HC) which represents a fusion between portions of donor and recipient chromosomes, while other portions of the participating chromosomes are lost (Figure 3C). HCs result from aberrant processing of BIR intermediates (Figure 2G, 2K, 2L). HCs are very frequent in Polδ mutants (*pol32Δ* and *pol3-ct*) that can successfully undergo the strand invasion step of BIR, but fail to initiate DNA synthesis [27,29]. This problem was suggested to promote the resolution of a HJ followed by a fusion between portions of the donor and recipient chromosomes. Symington’s group observed that Mus81 is responsible for approximately one half of HCs observed in *pol3-ct* background [29]. However, it is likely that other resolvase proteins also promote HCs, since the other half of the HCs occurred independently of *MUS81*. Although HCs are more frequent in mutants, they are also observed in wild-type cells [27,29], where HCs may have resulted from pausing of BIR-associated DNA synthesis (Figure 2G). 

An important feature of HCs is that they create a broken DNA end in a previously intact donor chromosome. This can lead to a cycle of instability where broken molecules will initiate rounds of HCs, resulting in the transfer of genetic instability from one molecule to another. This phenomenon is similar to cascades of non-reciprocal translocations (NRTs) described in mammalian tumors [98]. It is therefore possible that an interruption of BIR could lead to an outcome similar to the phenomenon of NRT in mammals. An increased level of HCs has also been observed in *rad51Δ* and *rad54Δ* mutants with a defect at the step of strand invasion [29,79]. This suggests that early state deficiencies of BIR can lead to HCs. Moreover, HCs were also observed in *rad52Δ* cells, however at very low rates [77,99]. To explain these observations, it has been suggested that when strand invasion is inefficient, HCs can form by SSA between long single-stranded DNA regions that were created by long resection of broken DNA ends.

## 8. Microhomology-Mediated BIR (MMBIR)

In recent years a new BIR-related pathway called micro-homology mediated BIR (MMBIR) has been proposed to explain copy number variations and complex chromosomal rearrangements including those associated with cancer and various other diseases in humans [100]. This idea was initiated by Lee *et al*. [101] who studied patients that had a duplication of the dosage-sensitive Proteolipid Protein 1 (*PLP1*) gene associated with Pelizaeus-Merzbacher disease (PMD) and discovered that many of these patients had complex DNA duplications and triplications. The authors analyzed the break-points of these rearrangements and found the presence of micro-homologies. They proposed the replication-based mechanism FoSTeS, adapted from Slack *et al*. [102], as a plausible mechanism capable of generating complex rearrangements via micro-homologies. They suggested that continual stalling of the replication fork could prompt disengagement of the nascent DNA strand which would allow this strand to switch to another active replication fork whereby micro-homology would be used to re-initiate synthesis (Figure 2H). 

The idea that CNVs result from a replication-based mechanism was further supported in budding yeast [103]. In this study the authors observed spontaneous segmental duplications (SDs) of the *RPL20B* gene. The authors demonstrated that SD formation was enhanced when replication was perturbed by *clb5* mutations or by the addition of camptothecin, a topoisomerase I inhibitor known to incite collapses of replication forks. The authors also observed that SD formation required Pol32, indicative of a BIR-based mechanism. Importantly, SDs were formed through Rad51-dependent and Rad51-independent pathways. The latter pathway proceeded via micro-homologies. 

As an attempt to provide an explanation for how CNVs and complex chromosomal rearrangements arise, Hastings *et al*. [100] proposed the model of MMBIR. According to this model, MMBIR is activated when Rad51 is absent or becomes limited during times of cellular stress (Figure 2A, 2H). They reasoned that the absence of Rad51 limits homologous invasion and allows DNA interactions using micro-homologies. In the MMBIR model, successive template-switching events result from DNA breakage that leads to repeated dissociation of single-stranded DNA in a replication fork, followed by re- annealing and re-initiation of the fork on different templates. 

Within the last few years, MMBIR has gained much attention in relation to the discovery of chromothripsis [104,105,106,107]. Chromothripsis is a phenomenon in which massive chromosomal rearrangements are typically restricted to one chromosome. The chromosome is first thought to ‘shatter’ resulting in extensive double-strand breaks, followed by random ‘stitching’ of the chromosomal fragments. Similar chromosome catastrophes have been observed in other high resolution genome analyses, for example by Liu *et al*. [108]. In this particular study, analyses from 17 individuals showed copy number variations including deletions, duplications, triplications, translocations and inversions that were all localized to one particular chromosome. Many of the chromosome alterations involved high copy number changes, with one individual having a total of 18 changes. The authors proceeded to sequence some of the break-point junctions in this individual and others, revealing frequent occurrences of templated insertions, and inversions with micro-homologies at break-points. They reasoned MMBIR as the most likely underlying mechanism responsible for generating these profound changes. Recently, MMBIR mechanisms have also been used to explain GCRs found in a number of genomic disorders in humans [101,109,110,111,112] and cancer [113]. Specifically, in-frame gene fusions of the RAF family protein kinases found in low-grade astrocytomas were strongly argued to be a consequence of MMBIR [113]. One significant aspect of this finding is that the in-frame gene fusions found in this study are also thought to be recurrent in various cancer types such as leukemias, lymphomas, and sarcomas [68], suggesting the possibility that these chromosomal rearrangements may develop via an MMBIR pathway. Interestingly, MMBIR is now being used to explain certain chromosomal rearrangements in plant organelle genomes. Recent studies in *Arabidopsis* have identified plastid-localized single-stranded DNA binding proteins called Whirly as important factors associated with suppressing micro-homology dependent recombination, which could be MMBIR [114].

Important questions regarding MMBIR include, which proteins are involved in promoting MMBIR and what cellular properties are required for this pathway to ensue. By a first approximation, it seems that replication stalling and/or replication collapse is necessary to prompt template switching. In terms of DNA architecture, it has been suggested that problems with the progression of a DNA replication fork could result from palindromic sequences, secondary structures (*i.e.*, cruciform, hairpin loops) repeats *etc* [101,103].

## 9. Conclusions (Remaining Questions and Future Prospects)

Despite the significant progress that has recently been made in studies of BIR, many aspects of BIR in eukaryotes still remain unknown and will need to be uncovered in the future. Future studies will be required to characterize the replication fork driving BIR and the mode of BIR-associated DNA synthesis. In particular, it will be important to identify and understand the role of DNA helicases and DNA polymerases participating in BIR. Also, other key replication-associated factors such as PCNA and the MMR complex need to be characterized in the context of BIR. In addition, future studies will also need to be developed to determine whether BIR is driven by a *bona fide* replication fork and whether the inheritance of newly-synthesized DNA is semi-conservative or conservative. In addition, a full spectrum of BIR-associated mutagenesis will need to be identified. Together, this information will provide insight into why BIR is highly mutagenic and why it is frequently associated with chromosomal rearrangements. It will also be very important to determine the mechanisms that suppress BIR at the stage of its initiation. Knowledge of these mechanisms will allow us to understand why normal eukaryotic cells repair DSBs by GC, a safer repair pathway, and how some cells shift towards the more destabilizing pathway of BIR.

With the recent interest in MMBIR, we imagine that in the next few years, MMBIR will also be better understood. Currently, no experimental evidence exists for the predictions made by Hastings *et al*. [100], that Rad51 deficiency promotes MMBIR, and therefore further studies will need to be conducted to better understand this connection. In addition, it will be important for future studies to determine whether normal BIR can switch to MMBIR and which specific factors regulate such a switch. Furthermore, the identity of specific proteins involved in MMBIR will further clarify the link between MMBIR and disease. 

To date, BIR is predominantly studied by introducing targeted DNA breaks that utilize non-sister chromatids as a template for repair. This situation differs from a chromosome break resulting from a replication collapse, a scenario where BIR can use a sister chromatid for repair. The use of enzymes producing site-specific DNA nicks [115,116,117] will enable studies of BIR that occur during S-phase replication. Also, it is critical that BIR is further investigated in higher eukaryotes, especially in mammals, where our knowledge of BIR mechanisms and the participating proteins remains obscure. Most importantly, future studies will need to shed light on the role of BIR in promoting mutations and chromosomal rearrangements that lead to various human diseases, including cancer.
